# High-Entropy Lead-Free Perovskite Bi_0.2_K_0.2_Ba_0.2_Sr_0.2_Ca_0.2_TiO_3_ Powders and Related Ceramics: Synthesis, Processing, and Electrical Properties

**DOI:** 10.3390/nano13222974

**Published:** 2023-11-19

**Authors:** Vasile-Adrian Surdu, Mariana-Andreea Marinică, Roxana-Elena Pătru, Ovidiu-Cristian Oprea, Adrian Ionuț Nicoară, Bogdan Ștefan Vasile, Roxana Trușca, Adelina-Carmen Ianculescu

**Affiliations:** 1 Department of Science and Engineering of Oxide Materials and Nanomaterials, Faculty of Chemical Engineering and Biotechnologies, National University of Science and Technology Politehnica Bucharest, Gheorghe Polizu 1-7, 011061 Bucharest, Romania; adrian.surdu@upb.ro (V.-A.S.); mariana.marinica@stud.chimie.upb.ro (M.-A.M.); adrian.nicoara@upb.ro (A.I.N.); 2 National Institute for Materials Physics, Atomistilor 405A, 077125 Magurele, Romania; roxana.patru@infim.ro; 3 Department of Inorganic Chemistry, Physical Chemistry and Electrochemistry, Faculty of Chemical Engineering and Biotechnologies, National University of Science and Technology Politehnica Bucharest, Gheorghe Polizu 1-7, 011061 Bucharest, Romania; ovidiu.oprea@upb.ro; 4 National Centre for Micro and Nanomaterials, National University of Science and Technology Politehnica Bucharest, Splaiul Independentei 313, 060042 Bucharest, Romania; bogdan.vasile@upb.ro (B.Ș.V.); roxana_doina.trusca@upb.ro (R.T.)

**Keywords:** high-entropy ceramics, perovskite, relaxor, Pechini method, Bi_0.2_K_0.2_Ba_0.2_Ca_0.2_Sr_0.2_TiO_3_

## Abstract

A novel high-entropy perovskite powder with the composition Bi_0.2_K_0.2_Ba_0.2_Sr_0.2_Ca_0.2_TiO_3_ was successfully synthesized using a modified Pechini method. The precursor powder underwent characterization through Fourier Transform Infrared Spectroscopy and thermal analysis. The resultant Bi_0.2_K_0.2_Ba_0.2_Sr_0.2_Ca_0.2_TiO_3_ powder, obtained post-calcination at 900 °C, was further examined using a variety of techniques including X-ray diffraction, Raman spectroscopy, X-ray fluorescence, scanning electron microscopy, and transmission electron microscopy. Ceramic samples were fabricated by conventional sintering at various temperatures (900, 950, and 1000 °C). The structure, microstructure, and dielectric properties of these ceramics were subsequently analyzed and discussed. The ceramics exhibited a two-phase composition comprising cubic and tetragonal perovskites. The grain size was observed to increase from 35 to 50 nm, contingent on the sintering temperature. All ceramic samples demonstrated relaxor behavior with a dielectric maximum that became more flattened and shifted towards lower temperatures as the grain size decreased.

## 1. Introduction

The ABX_3_-type perovskite structural family, first described by Goldschmidt in 1926 [1], continues to be a fascinating class of materials that has garnered significant attention in the field of materials science and engineering. These materials are renowned for their broad spectrum of multifunctional properties, including ferroelectricity, piezoelectricity, pyroelectricity, ferromagnetism, and nonlinear dielectric characteristics [2,3,4,5,6]. Such diverse properties make them suitable for a variety of applications, encompassing energy conversion and storage, sensors, filters, separators, detectors, antennas, and environmental remediation [7,8,9,10,11,12,13].

Among the perovskites, relaxor ceramics stand out due to their unique properties, which arise from disruptions in the lattice structure. This disruption leads to a deviation from the ideal perovskite structure, resulting in intriguing physical properties that are not observed in perfect perovskite structures [14]. A mandatory condition for the relaxor state is chemical disorder. Relaxors in binary systems show chemical disorder, especially on the *B*-site, *A*(*B*′_1−x_*B*_x_″)O_3_, but this heterogeneity can also be produced on the *A*-site, (*A*′_1−y_*A*″_y_)*B*O_3_, or even both *A* and *B* sites simultaneously, (*A*′_1−y_*A*″_y_)(*B*′_1−x_*B*_x_″)O_3_. Typical examples for *B*-site chemical non-homogeneity are Pb(Mg_1/3_Nb_2/3_)O_3_, Pb(Zn_1/3_Nb_2/3_)O_3_, Ba(Zr_1−x_Ti_x_)O_3_, Ba(Ti_1−x_Sn_x_)O_3_, and Ba(Ti_1−x_Ce_x_)O_3_ [15,16,17,18,19,20]. In the case of *A*-site heterogeneity, typical relaxors are (Bi_0.5_Na_0.5_)TiO_3_ and Bi_0.5_K_0.5_TiO_3_ [21,22], whereas for both *A*- and *B*-site chemical disorder, 0.8Pb(Mg_1/3_Nb_2/3_)O_3_-0.2PbTiO_3_ and (Ba_0.90_Ca_0.10_)(Zr_0.25_Ti_0.75_)O_3_ have been reported [23,24]. Even though cationic species of equivalent crystallographic sites are statistically distributed at a global scale, at a local scale, the order may vary between a quasi-total disorder to a low ordering degree. Chemical disorder leads to the breaking of long-range ordering with the gain in short-range ordering, where dipoles are still oriented but only in small regions, known as polar nanoregions (PNR) or clusters. Therefore, above the Curie temperature, *T*_C_, where the symmetry of the crystal structure corresponding to the paraelectric phase is cubic and no polarization is expected, nanoregions are formed and consist of a limited number of unit cells [25]. 

The primary strategies employed to enhance the relaxor behavior include chemical modifications at various crystallographic sites, the use of chemical additives that do not specifically target lattice sites, and microstructural design [26]. Recently, the concept of high entropy has emerged as a novel approach to material composition design. This concept, originally developed in the field of metallic materials, has since been extended to ceramics, including perovskites [27,28]. The configurational entropy ($S_{config}$) in perovskite ABX_3_ ceramics can be calculated using Equation (1) [29]:

(1) $$S_{config}=-R\left[{\left(\sum_{a=1}^{A}x_{a}\ln x_{a}+\sum_{b=1}^{B}x_{b}\ln x_{b}\right)}_{cation-site}+{\left(\sum_{j=1}^{M}x_{j}\ln x_{j}\right)}_{anion-site}\right]$$

where *A*, *B*, and *M* represent the number of element species at the *A*-site cation and *B*-site cation and anion sites, respectively; *x_a_*, *x_b_*, and *x_j_* represent the mole fraction of the corresponding elements; and *R* is the gas constant [29]. From this perspective, the most extensively researched relaxors are typically found in regions of low entropy (*S* < 1*R*) or medium entropy (1*R* < *S* < 1.5*R*). The term “high entropy” is reserved for compositions where the entropy (*S*) is at least 1.5 times the gas constant, *R*. This high-entropy state is typically achieved when there are at least five elements occupying the same site and their atomic fractions are equal. In addition, supplementary disorder factors on each of the crystallographic sites of the perovskite oxides may be calculated by Equations (2) and (3) [30]:

(2) $$\delta \left(R_{A}\right)=\sqrt{\sum_{i=1}^{N}c_{i}\left(1-\frac{R_{A_{i}}}{\sum_{i=1}^{N}c_{i}R_{A_{i}}}\right)}$$

(3) $$\delta \left(R_{B}\right)=\sqrt{\sum_{i=1}^{N}c_{i}\left(1-\frac{R_{B_{i}}}{\sum_{i=1}^{N}c_{i}R_{B_{i}}}\right)}$$

where $\delta \left(R_{A}\right)$ is the *A*-site cation-size difference, $\delta \left(R_{B}\right)$ is the *B*-site cation-size difference, $R_{A_{i}}$ is the radius of the *i*th cation on the *A*-site, $R_{B_{i}}$ is the radius of the *i*th cation on the *B*-site, and *c_i_* is the mole fraction of the *i*th cation. It is interesting to note that while high entropy alloys typically require an atomic size difference (*δ*) of no more than 6.5% to form single-phase structures [31], high-entropy perovskite oxides have been reported to accommodate atomic-size differences of approximately 25%, with no apparent correlation with stability. A more reliable indicator of the stability of perovskite structures is the Goldschmidt tolerance factor, denoted as *t*. This factor can be calculated by Equation (4) [30]:

(4) $$t=\frac{R_{A}+R_{O}}{\sqrt{2}\left(R_{B}+R_{O}\right)}$$

where *t* is the tolerance factor, $R_{A}$, $R_{B}$, and $R_{O}$ are the average radius on the *A*-site and *B*-site and the oxygen radius, respectively. For *t* > 1, a tetragonal structure is stable, for 0.9 < *t* < 1, a cubic structure is stable, and for 0.71 < *t* < 0.9, orthorhombic/rhombohedral structures are stable [1]. Stable high-entropy perovskite oxide compositions have been documented for the *A*-site [32,33,34,35,36,37,38,39,40,41,42,43,44], *B*-site [45,46,47,48], and both cation sites [49,50,51,52,53,54]. However, these studies do not consider charge balancing or compensation when heterovalent cations are mixed. As a result, the dielectric behavior of these compositions remains not fully understood. In the case of *A*-site high-entropy compositions with balanced charge, those with a tolerance factor *t* > 1 demonstrated high relative permittivity values, which are associated with the phase transition region from tetragonal to cubic [44]. However, these compositions contain 20% lead on the *A*-site of the perovskite structure (Table 1).

The aim of our study is to synthesize and characterize the dielectric behavior of a novel lead-free *A*-site high-entropy perovskite oxide, i.e., Bi_0.2_K_0.2_Ba_0.2_Sr_0.2_Ca_0.2_TiO_3_ (BiKBSCT), with balanced charge. In addition to a configurational entropy of $S_{config}>1.5R$, we considered the atomic size difference descriptor *δ*(*R_A_*) < 25% and a tolerance factor of *t* > 1 to ensure the stability of the perovskite structure and to encourage a morphotropic phase transition from a tetragonal to a cubic phase. The composition we established is compared using the aforementioned descriptors to the currently reported compositions in Table 1.

In selecting the synthesis method, our goal was to achieve a uniform particle size distribution for enhanced processability. From this perspective, the Pechini method stands out as superior to other methods such as solid-state, mechanochemical, spray pyrolysis, hydrothermal, or sol–gel methods, which have been reported in the synthesis of high-entropy perovskite oxides [58]. The Pechini method’s ability to produce a more consistent particle size distribution contributes to its effectiveness in synthesizing these complex materials. The successfully obtained powder was shaped into cylindrical samples and the related ceramics consolidated by conventional sintering were analyzed from structure, microstructure, and dielectric properties points of view.

## 2. Materials and Methods

### 2.1. Powder Synthesis

The (Bi_0.2_K_0.2_Ba_0.2_Sr_0.2_Ca_0.2_)TiO_3_ powder was synthesized using the Pechini route [59]. The initial step involved creating a metal citrate solution by dissolving bismuth nitrate pentahydrate (Bi(NO_3_)_3_∙5H_2_O, Sigma-Aldrich (St. Louis, MO, USA), ACS reagent, ≥98.0%), potassium nitrate (KNO_3_, Sigma-Aldrich, ACS reagent, ≥99.0%), barium nitrate (Ba(NO_3_)_2_, Sigma-Aldrich, ACS reagent, ≥99.0%), strontium nitrate (Sr(NO_3_)_2_, Sigma-Aldrich, ACS reagent, ≥99.0%), calcium nitrate tetrahydrate (Ca(NO_3_)_2_∙4H_2_O, Sigma-Aldrich, ≥99.0%), and citric acid (Sigma-Aldrich, ACS reagent, ≥99.5%) in water to maintain a molar ratio of 0.2:0.2:0.2:0.2:0.2:1 among the precursors.

The metal citrate solution was then combined with a clear yellowish solution of titanium isopropoxide, citric acid, and ethylene glycol in a 1:1 molar ratio between the total cations and citric acid and a 1:2 molar ratio between citric acid and ethylene glycol.

The resulting precursor solution was homogenized at 300 rpm and a temperature of 110 °C. After 4 h of stirring, the solution was transferred to an oven for polymerization at 110 °C for 48 h, yielding a yellowish-white precursor powder. The thermal behavior and phase composition of this precursor powder were analyzed before it was subjected to calcination at an appropriately chosen temperature.

### 2.2. Ceramics Processing

In the process of preparing the ceramics, green pellets with a diameter of 13 mm and an approximate thickness of 3 mm were produced through uniaxial pressing under a pressure of *P* = 180 MPa. Conventional sintering was carried out in air in a muffle furnace, at temperatures of 900 °C, 950 °C, and 1000 °C, respectively, with a plateau of 4 h and a heating rate of 5 °C/min. Afterward, the resulting ceramics were slowly cooled at room temperature and the samples were labeled as BiKBSCT-900, BiKBSCT-950, and BiKBSCT-1000.

### 2.3. Characterization

Infrared spectroscopic measurements were performed using the NICOLET 6700 FT-IR spectrophotometer (Thermo Electron Corporation, Waltham, MA, USA) with Fourier Transform (FT-IR) in transmission in the range of 400–4000 cm^−1^. The spectra were recorded on a thin, clear tablet (20 mg/cm^2^) of KBr containing approximately 0.5% of the sample. The pellets were prepared by the compaction and vacuum pressing of a homogeneous mixture obtained by grinding 1 mg of the substance in 200 mg KBr. For each sample, the spectra were recorded at a resolution of 4 cm^−1^ and processed using the OMNIC 7.3 software.

Thermogravimetric and differential thermal analyses (TG/DTA), using Mettler Toledo TGA/SDTA 851^e^ equipment (Greifensee, Switzerland), were used to assess the thermal behavior of the as-prepared samples in open Al_2_O_3_ crucibles and in flowing-air environments. The heating rate was 10 °C/min and the maximum temperature was set to 1300 °C.

Phase composition and structure were investigated using a PANalytical Empyrean X-ray diffractometer (Cedar Park, TX, USA) operated in theta–theta geometry. The instrument was equipped with a CuKα (λ = 1.5418 Å) sealed X-ray tube with a fixed 1/4° divergence slit and 1/2° anti-scatter slit on the incident beam side, and on the diffracted beam side, a 1/2° anti-scatter slit and a Ni-filter mounted on PIXCel3D detector operated in 1D mode. The analyses were conducted in the 10–80° 2θ range, with a step size of 0.026° and a counting time per step of 255 s. The recorded patterns were indexed using HighScorePlus 3.0.e software in conjunction with the Crystallography Open Database (COD). Rietveld refinement was carried out using a polynomial function for background approximation, a pseudo-Voigt function for peak profile, and a Caglioti function for peak width.

The local order of the samples was examined using Raman spectroscopy at room temperature. The instrument used for this analysis was a LabRAM HR Evolution spectrometer, manufactured by Horiba in Kyoto, Japan. The Raman spectra were obtained using the 514 nm line of an argon ion laser. The laser beam, with a power of 125 mW, was focused on spots of a few micrometers in size on the samples.

The morphology and microstructure of the (Bi_0.2_K_0.2_Ba_0.2_Sr_0.2_Ca_0.2_)TiO_3_ powder and corresponding ceramics processed in various conditions were investigated by scanning electron microscopy operated at 30 kV (Inspect F50, FEI, Hillsboro, OR, USA) and transmission electron microscopy operated at 300 kV (Tecnai^TM^ G2 F30 S-TWIN, FEI, Hillsboro, OR, USA). The average particle size of the (Bi_0.2_K_0.2_Ba_0.2_Sr_0.2_Ca_0.2_)TiO_3_ powder and the average grain size of the (Bi_0.2_K_0.2_Ba_0.2_Sr_0.2_Ca_0.2_)TiO_3_ ceramics were estimated from the value distributions, which were determined using the OriginPro 9.0 software (OriginLab, Northampton, MA, USA) by taking into account size measurements on ~100 particles/grains performed by means of the software of the electron microscopes (ImageJ 1.50b, National Institutes of Health and the Laboratory for Optical and Computational Instrumentation, Madison, WI, USA) in the case of SEM and Digital Micrograph 1.8.0 (Gatan, Sarasota, FL, USA) in the case of transmission electron microscopy (TEM).

Parallel plate capacitors with Ag-painted electrodes, for all (Bi_0.2_K_0.2_Ba_0.2_Sr_0.2_Ca_0.2_)TiO_3_ ceramic specimens, were configured for the electrical measurements. Dielectric spectroscopy measurements were carried out in a vacuum at temperatures between 100 and 500 K in the 10^2^–10^6^ Hz frequency range with a 0.5 V amplitude ac signal by using a HIOKI IM3536 impedance analyzer (Tokyo, Japan).

## 3. Results and Discussion

### 3.1. Powder Characterization

**Figure 1:**
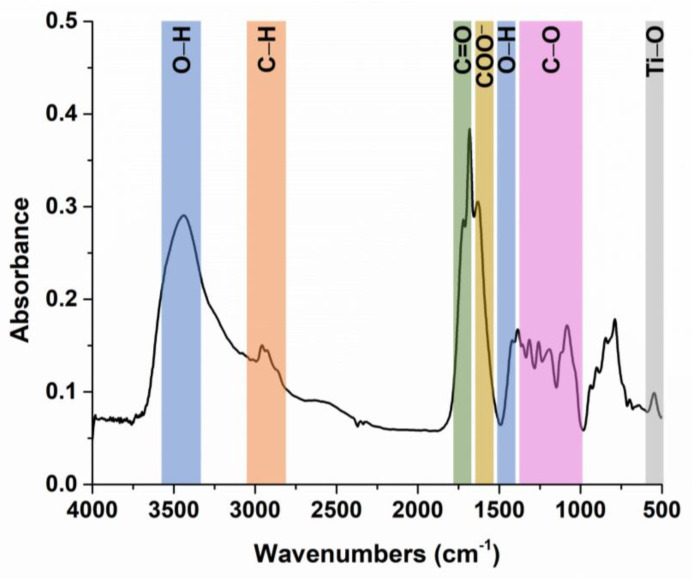
FT-IR spectrum of (Bi_0.2_K_0.2_Ba_0.2_Sr_0.2_Ca_0.2_)TiO_3_ precursor powder.

The powder obtained after the synthesis and drying process was investigated in terms of composition using infrared spectroscopy. The Fourier Transform Infrared (FT-IR) spectrum for the precursor powder is depicted in Figure 1.

The examination of the bands discerned from the FT-IR spectrum underscores the presence of various functional groups and their corresponding stretching and bending vibrations. These encompass the strong, broad O-H stretching band (3444 cm^−1^) corresponding to intermolecular groups, the medium stretching C-H band (2956 and 2925 cm^−1^) corresponding to alkyl groups, the strong stretching C=O band (1720 and 1682 cm^−1^) corresponding to carboxylic acid and conjugated acid groups, the strong COO^−^ band (1629 cm^−1^) corresponding to the carboxylate group, the medium bending O-H band (1419 cm^−1^) corresponding to the carboxylic acid group, the medium stretching C-O bands (1386, 1355, 1315, 1257, 1189, and 1118 cm^−1^) corresponding to carboxylic acid and ester groups, various bending bands associated with the 1081, 939, 902, 844, 788, 698 and 638 cm^−1^ wavenumbers, and the Ti-O band associated with the 547 cm^−1^ wavenumber. These bands are indicative of a tridentate metal complex formed by precursor metal ions, citric acid, and ethylene glycol.

**Figure 2:**
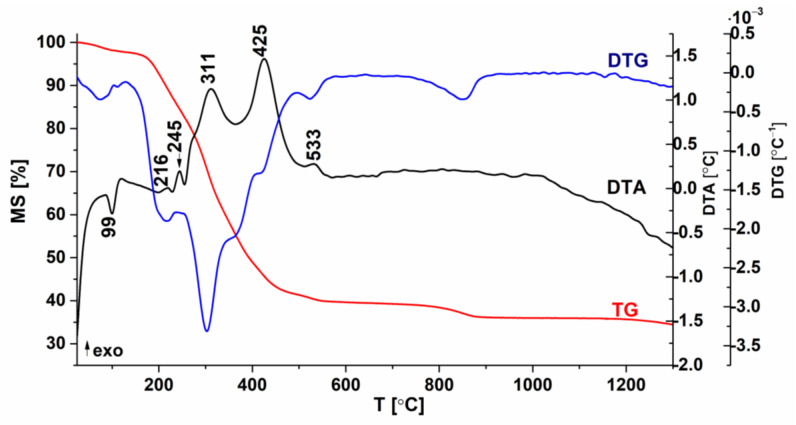
The TG (red)/DTG (blue)/DTA (black) curves of the (Bi_0.2_K_0.2_Ba_0.2_Sr_0.2_Ca_0.2_)TiO_3_ precursor powder.

The thermal stability of the precursor powder of (Bi_0.2_K_0.2_Ba_0.2_Sr_0.2_Ca_0.2_)TiO_3_ was investigated using thermogravimetric (TG)/differential thermogravimetric (DTG)/differential thermal analysis (DTA) in the temperature range of 20–1300 °C (Figure 2).

The powder exhibited a loss of residual moisture, accounting for 2.09% of its mass, up to temperatures of 200 °C, with the endothermic effect displaying a minimum at 99 °C. The powder underwent thermal decomposition, resulting in a mass loss of 58.08% in five stages, each characterized by exothermic maxima at temperatures of 216, 245, 311, 425, and 533 °C. Between the temperature range of 700–900 °C, an additional mass loss of 5.47% was observed, most likely due to some volatilization processes related to Bi^3+^ and K^+^ species. The residual mass, which appeared as a white-yellowish powder, constituted 34.36% of the original mass. Thus, the calcination of the precursor powder was performed at 900 °C.

**Figure 3:**
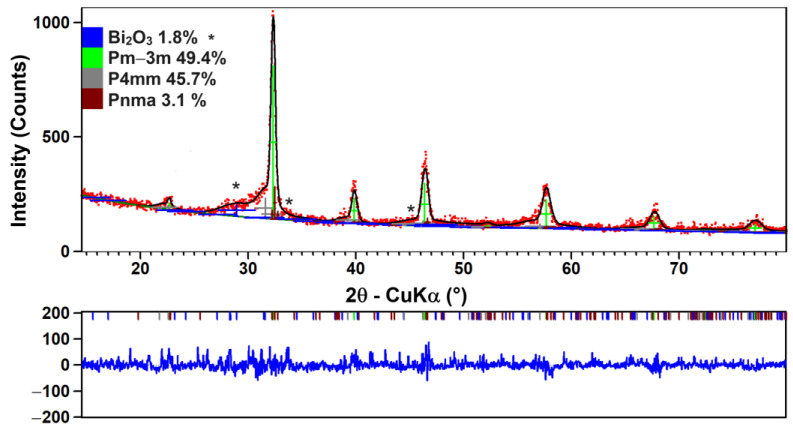
Rietveld refinement of the (Bi_0.2_K_0.2_Ba_0.2_Sr_0.2_Ca_0.2_)TiO_3_ calcined powder X-ray diffraction pattern.

**Figure 4:**
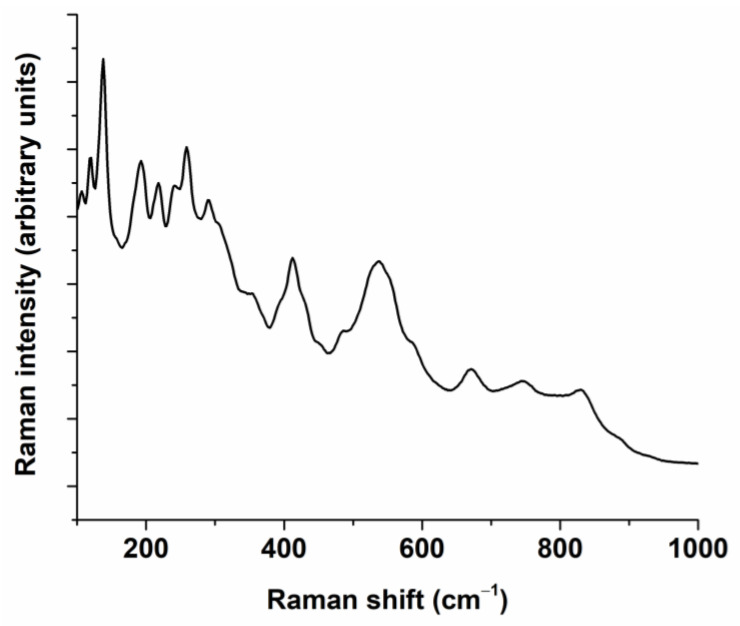
Raman spectrum specific to (Bi_0.2_K_0.2_Ba_0.2_Sr_0.2_Ca_0.2_)TiO_3_ calcined powder.

The powder obtained through calcination at 900 °C was further examined from a phase composition and structure point of view through the Rietveld refinement of the XRD pattern (Figure 3). As depicted in Figure 3, the main diffraction maxima exhibit an asymmetric Gaussian profile, suggesting the presence of a mixture of perovskite polymorphs. This is further corroborated by the emergence of a split maximum in the Raman spectrum (Figure 4) within the 200–400 cm^−1^ range. Besides a mixture of polymorphs, the broad peak widths suggest a low crystallite size.

The Rietveld refinement of the XRD pattern specific to the (Bi_0.2_K_0.2_Ba_0.2_Sr_0.2_Ca_0.2_)TiO_3_ powder was conducted considering each probable polymorphic form or their mixtures. The optimal refinement indices (*R*_exp_ = 17.17, *R*_p_ = 9.21, *R*_wp_ = 14.43, and *χ*^2^ = 0.71) were achieved when a mixture of cubic, tetragonal, and orthorhombic polymorphs, along with a secondary phase of Bi_2_O_3_, was considered. Details regarding the phase proportion, unit cell parameters, average crystallite size of the perovskite polymorphs, and refinement indices can be found in Table 2.

The main identified phases consist of a 49.7% perovskite phase with cubic Pm-3m symmetry (COD #96-900-6865 [60]), a 45.7% perovskite phase with tetragonal P4mm symmetry (COD #96-151-3253 [61]), a 3.1% distorted perovskite phase with Pnma symmetry (COD #96-900-2804 [62]), and a low amount of 1.8% of *δ*-Bi_2_O_3_ secondary phase (COD #96-101-0313). As suggested by the peak width broadening, the average crystallite size is low and was assessed as follows: 14.24 ± 9.12 nm for the cubic perovskite phase, 1.93 ± 0.26 nm for the tetragonal perovskite phase, and 25.66 ± 6.23 nm for the orthorhombic perovskite phase.

To further confirm the local structure of the (Bi_0.2_K_0.2_Ba_0.2_Sr_0.2_Ca_0.2_)TiO_3_ powder, Raman spectroscopy was performed on the sample. Figure 4 presents the specific Raman spectrum of (Bi_0.2_K_0.2_Ba_0.2_Sr_0.2_Ca_0.2_)TiO_3_ powder calcined at 900 °C.

Five types of vibrational modes were observed. The modes below ≈200 cm^−1^ correspond to A-O-type bonds. The existence of several modes in this frequency range shows the coexistence of various elements (Bi, K, Ba, Ca, Sr) and several polymorphic forms in accordance with previous studies [38,63,64,65,66]. The Raman modes within the range of 200–400 cm^−1^ are characteristic of Ti-O vibrations [65,66]. The modes between 400 and 700 cm^−1^ are representative of lattice distortion due to TiO_6_ octahedral vibration and can be separated in two peaks in the (Bi_0.2_K_0.2_Ba_0.2_Sr_0.2_Ca_0.2_)TiO_3_ powder [67]. For the range beyond 700 cm^−1^, the modes can be assigned to the behavior of oxygen vibration/rotation [68]. Thus, the Rietveld refinement of the XRD pattern and Raman spectrum reveal that perovskite polymorphs of cubic, tetragonal, and orthorhombic symmetry coexist.

The elemental chemical analysis of the (Bi_0.2_K_0.2_Ba_0.2_Sr_0.2_Ca_0.2_)TiO_3_ powder obtained from the X-ray fluorescence spectrum (Table 3) indicates the presence of constituent chemical elements of the powder (Ti, Bi, Ba, Sr, Ca, and K) in a proportion of 97.77%, along with secondary elements (F, Pd, Na, Pt, Al, V, Si, S, Mo, Hf, Ir, Y, Cl, Br, Ga, P, Fe) constituting 2.24%. The estimated formula for the compound derived from these data is (Bi_0.18_K_0.22_Ba_0.18_Sr_0.18_Ca_0.24_)TiO_3_.

**Figure 5:**
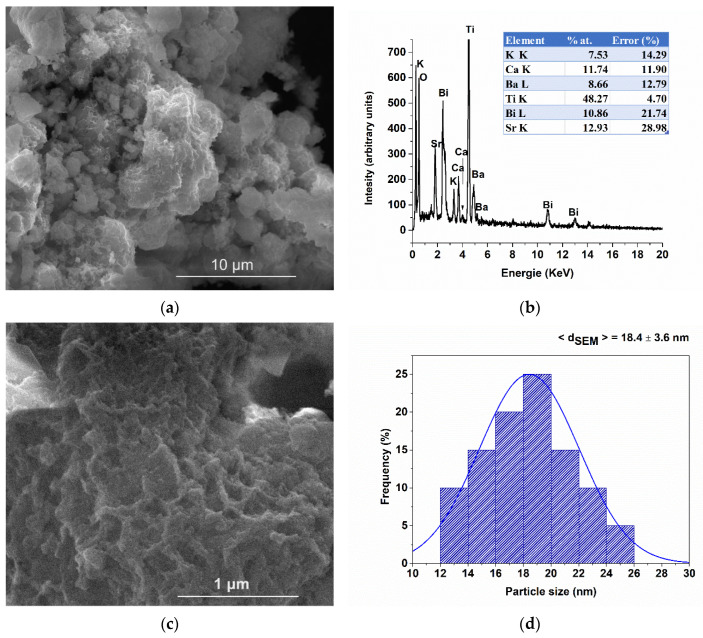
Overall SEM images (**a**) and detail (**c**), EDS spectrum (**b**) and particle size distribution (**d**).

The local morphology and chemical composition were examined using scanning electron microscopy (SEM) and transmission electron microscopy (TEM) in conjunction with energy-dispersive X-ray spectrometry (EDS). The secondary electron SEM image (Figure 5a) depicts spherical, porous aggregates, which are formed as a consequence of the decomposition of the precursor gel during the calcination heat treatment.

The chemical formula estimated from the EDS spectrum (Figure 5b), corresponding to the analyzed micro-area is (Bi_0.22_K_0.15_Ba_0.17_Sr_0.26_Ca_0.24_)TiO_3_, which is within the error limits of the analysis compared to the nominal formula. The detailed SEM image (Figure 5c) showcases the polyhedral, edge-rounded morphology of the constituent particles, which have an average size of 18.4 ± 3.6 nm and exhibit a narrow, unimodal size distribution.

**Figure 6:**
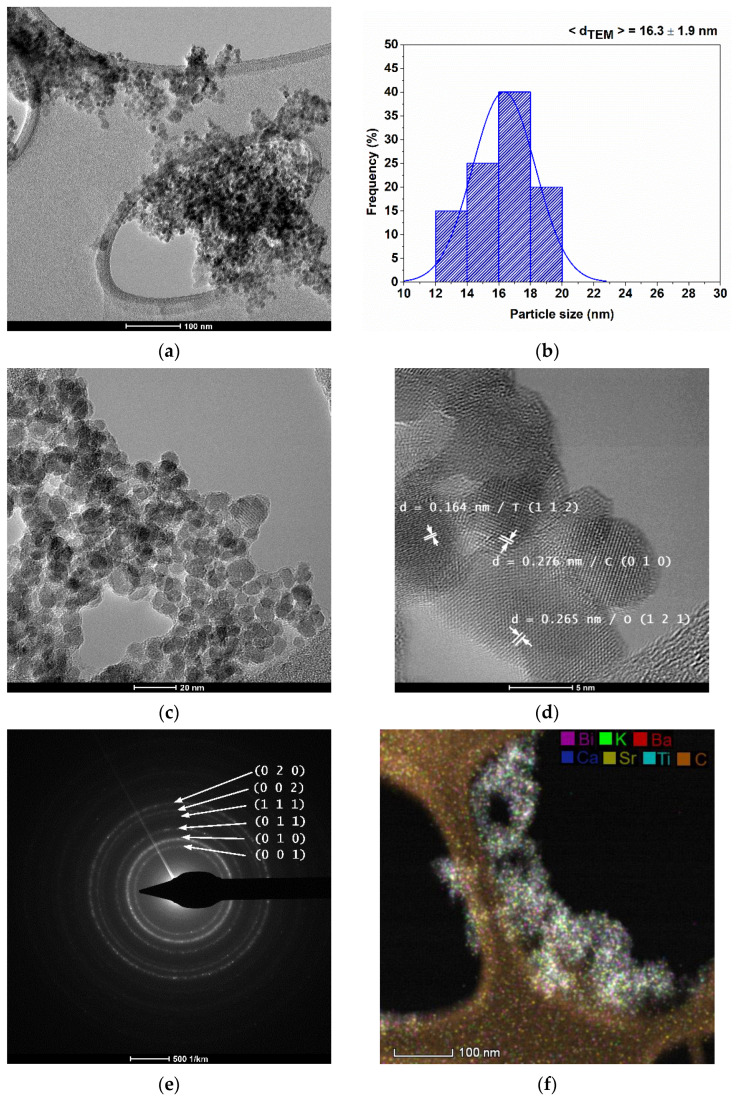
Bright-field TEM images (**a**,**c**), particle size distribution (**b**), high-resolution TEM image (**d**), selected area electron diffraction (**e**), and HRSTEM-EDS mapping (**f**).

Bright-field TEM images (Figure 6a,c) reveal aggregates of nanoparticles, each up to 20 nm in size, exhibiting a polyhedral morphology with rounded edges. The average particle size, determined to be 16.3 ± 1.9 nm with a narrow distribution (Figure 6b), as estimated from TEM measurements, aligns closely with the value obtained from SEM measurements and the average crystallite size derived from diffractometric data. This suggests the single-crystal nature of the particles.

The high-resolution TEM image (Figure 6d) depicts an aggregate of particles with rounded polyhedral shapes of varying polymorphic forms. The measured interplanar spacings of 0.164 nm, 0.276 nm, and 0.265 nm correspond to the tetragonal polymorph with the identified crystallographic plane (1 1 2), cubic polymorph with the identified crystallographic plane (0 1 0), and orthorhombic polymorph with the identified crystallographic plane (1 2 1), respectively.

The electron diffraction on the selected area (Figure 6e) is characteristic of a perovskite material with tetragonal symmetry, for which the crystal planes (0 0 1), (0 1 0), (0 1 1), (0 0 2), and (0 2 0) were identified. The diffraction rings are diffuse, indicative of nanostructured polycrystalline materials.

The HRSTEM-EDS mapping presented in Figure 6f underscores the homogeneous distribution of the constituent elements of the nanoparticles (Bi, K, Ba, Sr, Ca, and Ti) within areas associated with the nanoparticles on the TEM grid.

### 3.2. Ceramics Characterization

**Figure 7:**
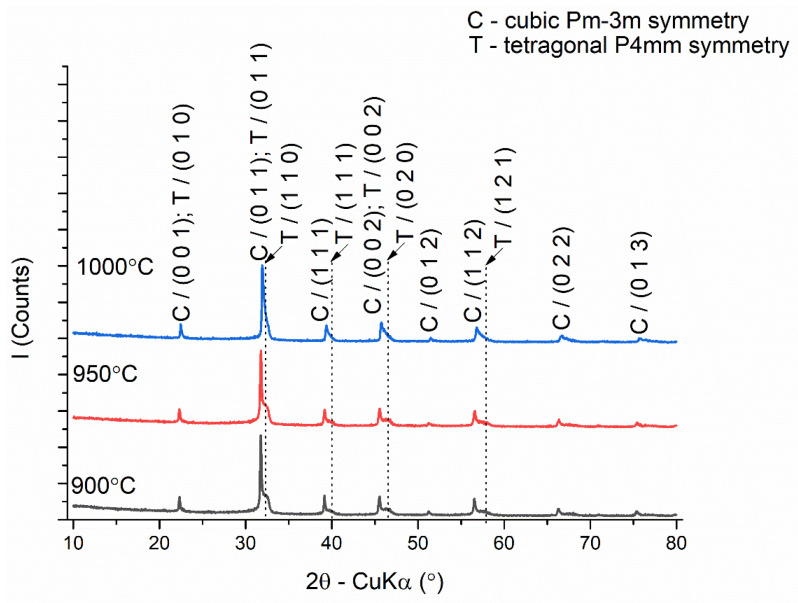
X-ray diffractograms of ceramic samples obtained by conventional sintering at temperatures of 900, 950, and 1000 °C, 4 h step.

The processing parameters were correlated with the phase composition evaluated from the X-ray diffraction data of the ceramic samples obtained by sintering under different conditions (Figure 7).

Figure 7 depicts diffraction patterns with asymmetric maxima for which the asymmetry decreases with the increase in the sintering temperature from 900 to 1000 °C, which indicates an increase in the compositional homogeneity of the perovskite phase. Crystal phase identification and Rietveld refinement of the diffraction patterns revealed a phase composition consisting of a cubic symmetry perovskite, space group Pm-3m (COD #96-900-6865 [60]), and a tetragonal symmetry perovskite (COD #96-151-3253 [61]), space group P4mm (Table 3).

The proportion of tetragonal phase increases from 54.3% to 73.7% with increasing sintering temperature from 900 to 1000 °C. This is accompanied by a decrease in the average crystallite size of the cubic phase from 40.31 ± 14.05 nm to 28.53 ± 15.25 nm and an increase in the average crystallite size of the tetragonal phase from 5.58 ± 2.23 nm to 7.93 ± 3.84 nm. The degree of tetragonality c/a of the tetragonal lattice decreases from 1.0380 to 1.0005 by increasing the unit cell parameter *a* and decreasing the value of the unit cell parameter *c* with the increase in the sintering temperature. The crystallographic density (ρ_t_) calculated using the weighted averaging of the phase fractions, the apparent density (ρ_a_) of the samples determined by volume displacement, and the relative density (ρ_r_) are presented in Table 4 versus sintering temperature. The somewhat unexpected lower densification of the BiKBSCT ceramics sintered at higher sintering temperatures can be associated with volatilization processes involving Bi^3+^ and K^+^ species. This assumption is in agreement with the thermal analysis (DTG and TG) data. The agglomeration of the as-generated cation vacancies in the grain boundary regions results in a higher intergranular porosity which prevents a more intensive grain growth process.

**Figure 8:**
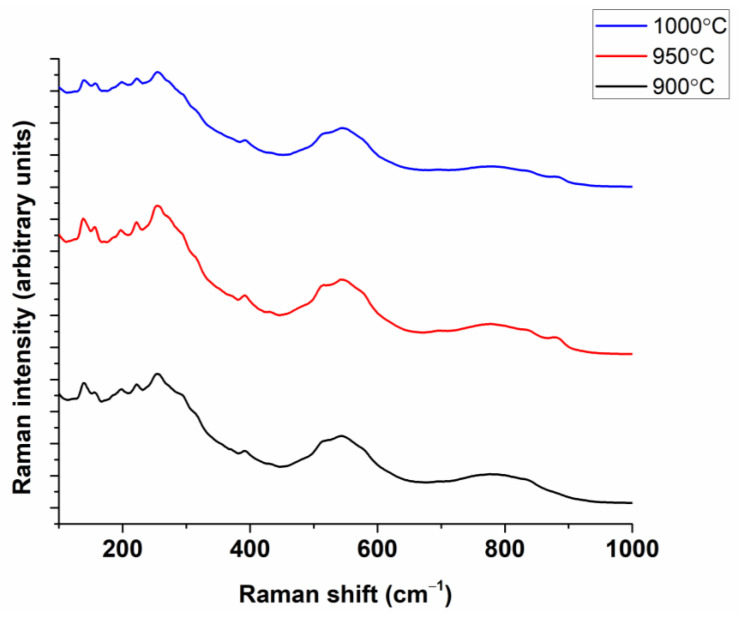
Raman spectra corresponding to (Bi_0.2_K_0.2_Ba_0.2_Sr_0.2_Ca_0.2_)TiO_3_ ceramics prepared by conventional sintering.

The local structure of the (Bi_0.2_K_0.2_Ba_0.2_Sr_0.2_Ca_0.2_)TiO_3_ ceramics was further confirmed by Raman spectroscopy. Figure 8 presents the specific Raman spectrum of (Bi_0.2_K_0.2_Ba_0.2_Sr_0.2_Ca_0.2_)TiO_3_ ceramics processed by conventional sintering at different temperatures. The coexistence of five cations on the *A*-site of the perovskite structure leads to a chemical disorder state and, consequently, to the broadening of the Raman bands for (Bi_0.2_K_0.2_Ba_0.2_Sr_0.2_Ca_0.2_)TiO_3_ high-entropy ceramics compared to typical perovskites based on BaTiO_3_ [65] but which are similar to those reported for Bi_0.5_K_0.5_TiO_3_-based ceramics [69]. The Raman bands observed below 200 cm^−1^ correspond to *A*-O bonds [70] including the bands of Bi-O, K-O, Ba-O, Ca-O, and Sr-O [64]. The peaks occurring between 200 and 400 cm^−1^ are related to the vibration of the Ti-O band occurring in both tetragonal and cubic symmetries also observed by Suchanicz et al. in the case of Na_0.5_Bi_0.5_TiO_3_−BaTiO_3_ ceramics [64]. The mode between 400 and 700 cm^−1^ is representative of TiO_6_ octahedron vibration [69]. For the range exceeding 700 cm^−1^, the modes can be attributed to the vibration/rotation of oxygen [68].

**Figure 9:**
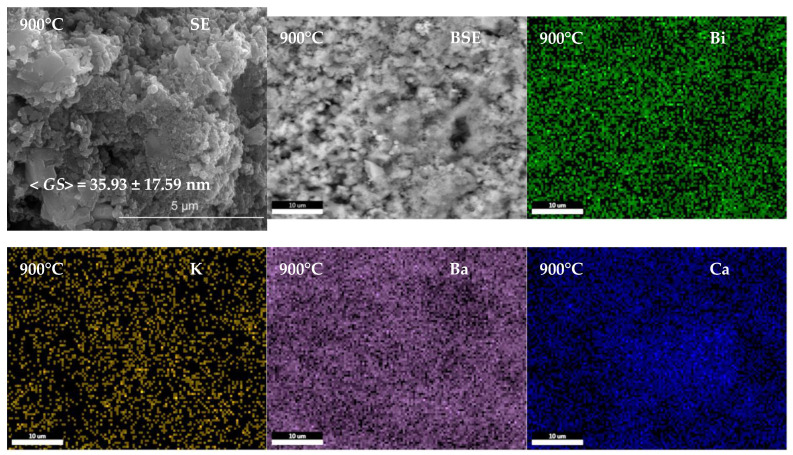

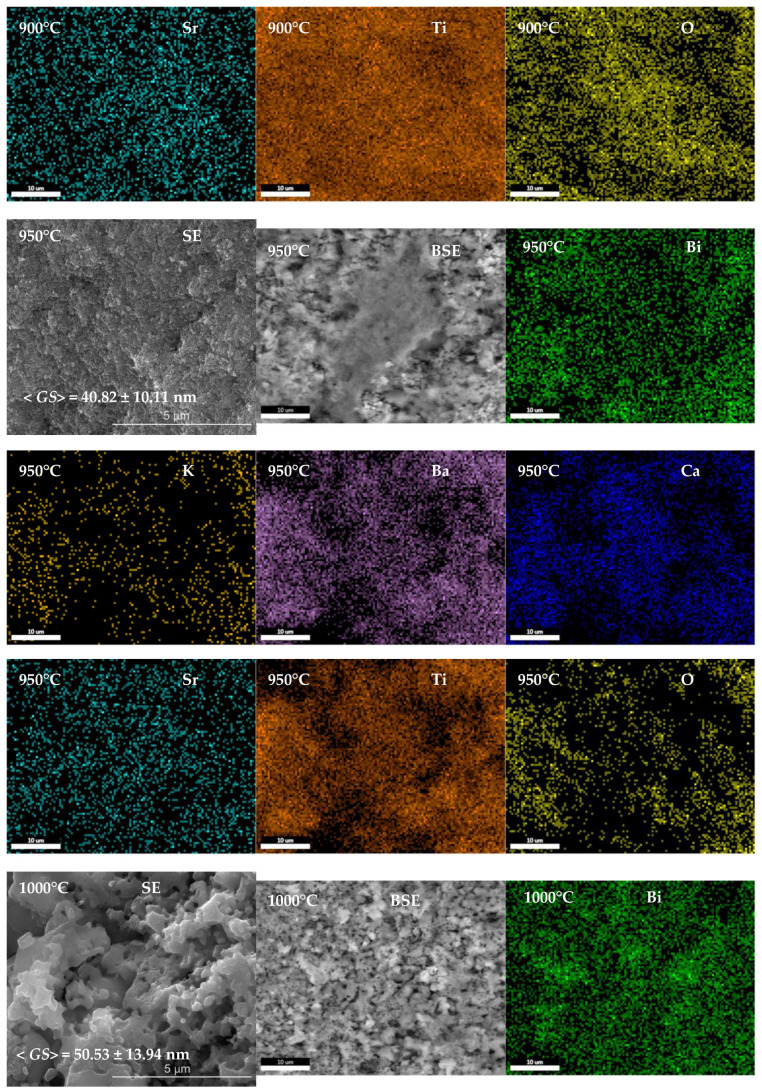

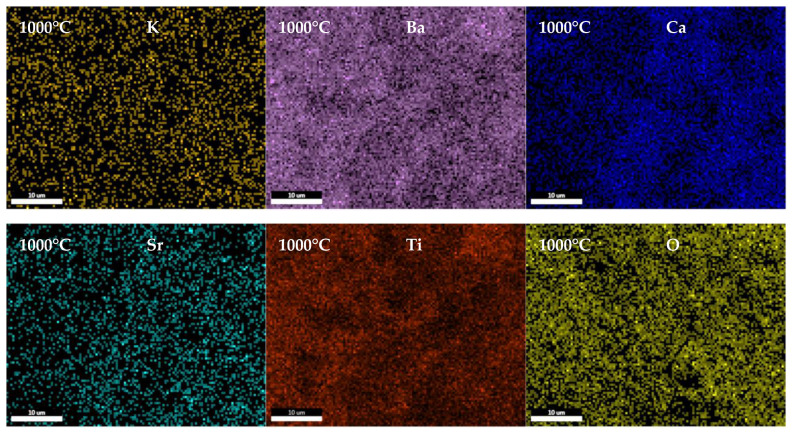
Secondary electron SEM images (SE), backscattered electron SEM images (BSE), and corresponding energy-dispersive X-ray spectrometry images of Bi, K, Ba, Ca, Sr, Ti, and O elements for (Bi_0.2_K_0.2_Ba_0.2_Ca_0.2_Sr_0.2_)TiO_3_ ceramics sintered at 900, 950, and 1000 °C.

The microstructure and microcomposition of the consolidated ceramic materials were analyzed by scanning electron microscopy and EDS mapping by viewing the cross-section (Figure 9). Backscattered electron images and EDS maps highlight a homogeneous distribution of chemical elements in the obtained materials. The ceramic materials are densified, showing intergranular porosity, and are nanostructured, showing polyhedral, rounded grains with sizes increasing from 35.93 ± 17.59 nm to 50.53 ± 13.94 nm as the sintering temperature increases from 900 to 1000 °C.

**Figure 10:**
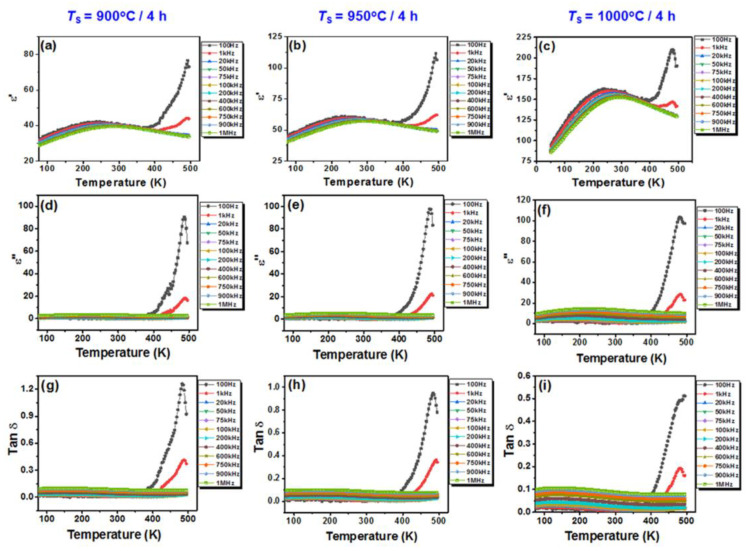
Temperature dependence of (**a**–**c**) the real part of the permittivity *ε*′, (**d**–**f**) the imaginary part of the permittivity *ε*″, and (**g**–**i**) the dielectric losses (tan*δ*) for the high-entropy BiKBSCT ceramics consolidated by conventional sintering at different temperatures: (**a**,**d**,**g**) 900 °C; (**b**,**e**,**h**) 950 °C; and (**c**,**f**,**i**) 1000 °C.

The temperature dependence of the real part and imaginary part of the dielectric permittivity, as well as of the tangent of the loss angle corresponding to the ceramics sintered by a conventional method, is shown in Figure 10.

Regardless of the sintering temperature, the temperature dependence of the real part of the permittivity shows permittivity maxima, *ε*′_m_, in the temperature range between 250 and 300 K, depending on the frequency (Figure 10a–c). “Diffuse” permittivity maxima, together with the frequency dispersion of the dielectric response at temperature values below the temperature of the permittivity maximum, *T*_m_, and the shift of *T*_m_ towards higher temperature values with increasing frequency, are characteristics associated with a relaxor state. However, the typical relaxors also show high values of a few thousand for the permittivity maxima, *ε*_m_ [71,72].

**Figure 11:**
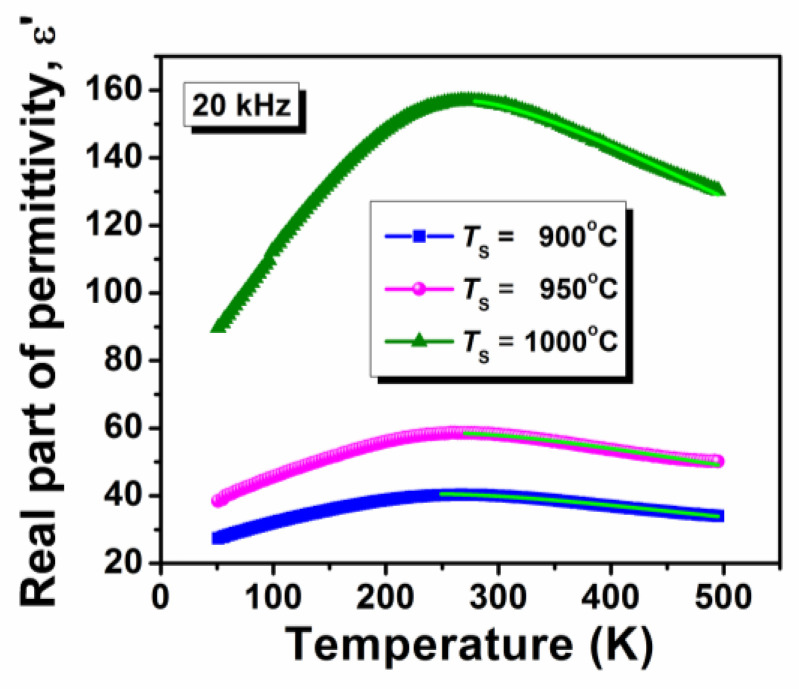
The temperature dependence of the real part of the permittivity *ε*′(*T*) recorded at a frequency of 20 kHz for the high-entropy ceramics consolidated by conventional sintering at different temperatures (the green lines represent the fits based on the modified Curie–Weiss equation provided by Santos et al. [81,82]).

For the nanocrystalline BiKBSCT samples under investigation, the values of the permittivity maximum vary depending on the grain size induced by the sintering temperature. As the sintering temperature increases from 900 to 1000 °C, the permittivity maximum of the ceramics increases from 41 to 160 at 1 kHz and from 39 to 152 at a frequency of 1 MHz. The low values of the permittivity maxima can be explained in terms of downscaling the grain size in the nanometer range, irrespective of the sintering temperature. Thus, as the grain size decreases from 50.53 nm for ceramics sintered at 1000 °C to 35.93 nm for ceramics sintered at 900 °C, the increasingly higher stress induced by internal micro-strains results in an increasing flattening of the permittivity maximum and the shift of the corresponding *T*_m_ towards lower temperature values (Figure 11). These features related to the so-called “grain size effect” were also observed in nanocrystalline BaTiO_3_ ceramics [73,74,75,76,77] as well as in related solid solutions such as Ba_0.85_Ca_0.15_Ti_0.9_Zr_0.10_O_3_ [78].

Perovskites with high *A*-site configurational entropy and with values of permittivity maxima between 700 and approximately 4500 have been reported in the literature [33,41,44,79]. However, in the reported studies, the ceramic materials were obtained by the solid phase reaction method, and the values of the average grain size ranged from 0.68 μm to 5–10 μm. Fang et al. [33] reported an increase in dielectric maximum temperature with increasing configurational entropy in Mn-doped (Bi_0.2_Na_0.2_Ca_0.2_Sr_0.2_Ba_0.2_)TiO_3_ ceramics. Following the values of the permittivity maxima reported in that study, it was concluded that they are the result of a competition between two factors acting with opposite effects, i.e., the high entropy and the grain size. The evolution versus temperature of both the imaginary part of the permittivity, *ε*″ (Figure 10d–f), and the loss angle tangent, tan *δ*, is quite similar (Figure 10g–i). Low values of the dielectric losses (tan*δ* < 8.5 × 10^−3^) were recorded at room temperature, especially for the samples sintered at 900 and 950 °C (Table 5). The surprisingly higher value (with about one order of magnitude) of tan *δ* at room temperature for the sample sintered at 1000 °C, exhibiting a slightly higher grain size relative to that of the samples sintered at 900 and 950 °C, could be associated with the lower densification of this specimen. The steep increases in *ε*′ (Figure 10a–c), *ε*″ (Figure 10d–f), and tan*δ* (Figure 10g,i) at temperatures above 300 K in the low-frequency range (below 20 kHz) are associated with interfacial Maxwell–Wagner phenomena [80].

In order to better describe the character and “diffuseness” degree of the phase of the nanocrystalline BiKBSCT ceramics under investigation, a modified Curie–Weiss law (Equation (5)) was used to fit the experimental data in the paraelectric region (Figure 11) [81,82].

(5) $$\varepsilon \prime =\frac{{\varepsilon \prime }_{m}}{1+{\left(\frac{T-T_{m}}{\Delta }\right)}^{\xi }},$$

where *ξ* is a parameter that indicates the ferroelectric–relaxor crossover, i.e., *ξ* = 1 corresponds to a typical ferroelectric while *ξ* = 2 is specific to a full relaxor character, and Δ is a parameter that defines the degree of the diffuseness of the ferroelectric phase transition. For the high-entropy ceramics under investigation, the fits (green lines in Figure 11) of the experimental permittivity data recorded at a frequency of 20 kHz in the paraelectric phase led to intermediate *ξ* values, between 1 and 2 (Table 5), revealing a ferroelectric–relaxor crossover. The grain size decrease favors the relaxor character reflected in the higher values of the two *ξ* and Δ parameters (Table 5), which means that the interaction between the polar nanoclusters has to be taken into account.

**Figure 12:**
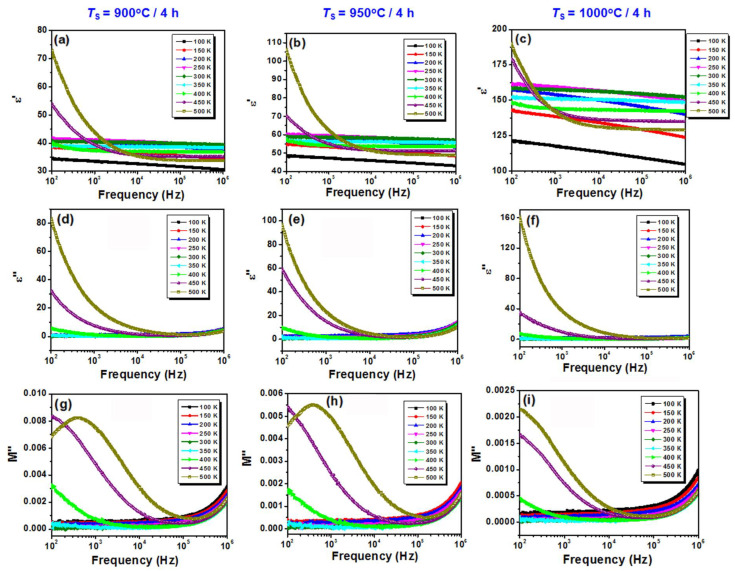
Frequency dependence of (**a**–**c**) the real part of the permittivity *ε*′, (**d**–**f**) the imaginary part of the permittivity *ε*″, and (**g**–**i**) the imaginary part of the dielectric modulus *M*″ for the high-entropy BiKBSCT ceramics consolidated by conventional sintering at different temperatures: (**a**,**d**,**g**) 900 °C; (**b**,**e**,**h**) 950 °C; and (**c**,**f**,**i**) 1000 °C.

The interfacial relaxation associated with the Maxwell–Wagner phenomena at higher temperatures (above 450 K) in the low-frequency region is clearly emphasized by the frequency dependence of *ε*′ (Figure 12a–c) and *ε*″ (Figure 12d–f). Thereby, the steep increase in the high-temperature dielectric response at frequency values below 10^3^ Hz seems to represent the downward branch of this relaxation process. For temperatures below 450 K, the dielectric response is almost frequency invariant, especially for the slightly denser BiKBSCT-900 and BiKBSCT-900 ceramics (Figure 12a–f).

From the complex dielectric modulus, M*, defined as [77]:

(6a) $$M^{*}\left(f\right)=M^{\prime }\left(f\right)+M^{\prime \prime }\left(f\right)$$

where

(6b) $$M^{\prime }\left(f\right)=\frac{\varepsilon^{\prime }\left(f\right)}{{\varepsilon^{\prime }}^{2}\left(f\right)+{\varepsilon^{\prime \prime }}^{2}\left(f\right)}$$

and

(6c) $$M^{\prime \prime }\left(f\right)=\frac{\varepsilon^{\prime \prime }\left(f\right)}{{\varepsilon^{\prime }}^{2}\left(f\right)+{\varepsilon^{\prime \prime }}^{2}\left(f\right)}$$

of particular interest is its imaginary part, *M*″, since it provides useful information about the charge transport mechanisms, such as electrical transport and conductivity relaxations in ceramics. It is well-known that the dielectric relaxation phenomena give maxima in both the *ε*″(*f*) and *M″*(*f*) dependences, whereas conductivity relaxations show maxima only in *M″*(*f*) spectra. In the case of the BiKBSCT ceramics under investigation, due to the presence of maxima in both *ε*″(*f*) and *M*″(*f*) spectra in the low-frequency range, one can conclude that only interfacial relaxation phenomena occur. The values of the *M*″(*f*) maxima increase and are shifted towards higher frequencies, varying from a value below 100 Hz to frequency values of ~400 Hz as the grain size decreases (Figure 12g–i).

**Figure 13:**
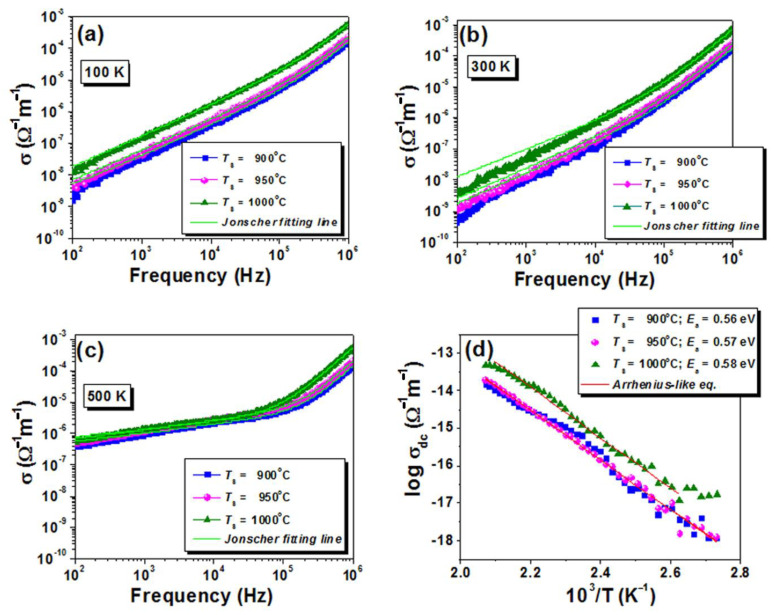
Frequency dependence of the ac conductivity for the ceramics conventionally consolidated in different conditions at some fixed measuring temperatures: (**a**) 100 K; (**b**) 300 K; (**c**) 500 K (the green lines represent the fits based on Jonscher’s equation). (**d**) Arrhenius plots of the dc conductivity.

Figure 13a–c shows the frequency dependence of the conductivity measured for the high-entropy ceramics at three representative temperatures of 100 K, 300 K, and 500 K. The frequency-dependence ac conductivity is characterized by an inflection region separating two frequency ranges dominated by different hopping conduction mechanisms within the grain boundaries of the material. At temperatures below room temperature (Figure 13a), the inflection is less marked and the conductivity arises from the charge carriers jumping between the localized states. Furthermore, with increasing temperature, the two frequency ranges in which the ac conductivity varies differently with increasing frequency are more clearly defined (Figure 13b,c). Thus, the two regions are clearly separated at ~105 Hz at a measuring temperature of 500 K (Figure 13c). Such an evolution with increasing frequency is best described by the hopping relaxation model, in which short-range hopping motions of polarons contribute to the high-frequency conductivity (the term *Bω^m^*) and the long-range translational hopping mechanism in the low-frequency range is associated with the *Aω^n^* term and approaches the direct current conductivity *σ_d.c._* [83,84,85,86].

(7) $$\sigma_{ac}\left(\omega \right)=\sigma_{0}+A\omega^{n}+B\omega^{m}$$

where $\sigma_{ac}\left(\omega \right)$ is the total conductivity at the ω angular frequency, $\sigma_{0}$ is the is the frequency-independent dc conductivity, which can also be expressed as $\sigma_{dc}$, *A* and *B* represent pre-exponential factors characterizing the material, and the *n* (0 ≤ *n* ≤ 1) and *m* (0 ≤ *m* ≤ 2) exponents describe the two different regions of frequency dependence of conductivity.

The parameters obtained by fitting the ac conductivity vs. frequency plots at temperatures below room temperature (RT) (Figure 13a,b) suggest that at lower temperatures, charge carriers exhibit almost independent hopping behavior with a weaker correlation between hops. The conduction is closer to an ideal or “classical” hopping process, where each hopped charge carrier is relatively independent of the others and the *n* exponent takes values close to 1. With increasing thermal energy, the charge carriers jump more easily over potential barriers, and their motion is also more influenced by interactions with the others and characterized by a slow relaxation when *n* takes lower values (see Figure 13c and Table 6). This may lead to a different type of conduction mechanism, i.e., a thermally activated process. The second exponent, *m*, in the double power law indicates the nature of the dispersion at a high frequency. Irrespective of temperature, conductivity is dominated by the rapid relaxation of charge carriers. Parameter *m* is indicative of a dipole relaxation process relatively insensitive to temperature variations, caused by the orientation and reorientation of dipoles in response to the alternating electric field.

Figure 13d shows the Arrhenius-like dependence of the conductivity with increasing temperature. The calculated activation energy values are quite similar in the range of 0.56–0.58 eV for all the samples, which seems to indicate the prevalence of the formation of single-ionized oxygen vacancies (V_O_**^·^**) [87,88,89].

**Figure 14:**
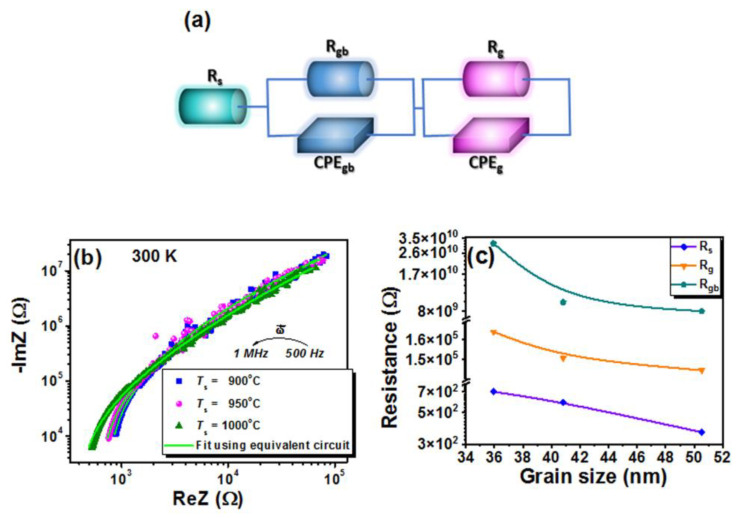
(**a**) Equivalent circuit used to fit the experimental impedance data, (**b**) Nyquist plots at 300 K on high-entropy ceramics (green line represents the fitting line), and (**c**) resistances calculated based on the equivalent circuit vs. the average grain size of the BiKBSCT ceramics.

Charge transport phenomena and thermally activated processes can be integrated and correlated at the macroscopic level with microstructural characteristics by the instrumentality of the electrochemical impedance spectroscopy. The physical processes represented by typical circuit elements in the equivalent circuit depicted in Figure 14a create a complete picture and a better understanding of the conduction and polarization mechanisms within the material. The equivalent circuit is designed to describe the two distinct semicircles that overlap to form a single entity with a wide area in the Re*Z* vs. -Im*Z* data (see Figure 14b). The resistance variation due to grain boundaries or structural heterogeneity leads to a large extension of Re*Z* in the real axis, while capacitances and polarization processes contribute to the height of the -Im*Z* in the imaginary axis. -Im*Z* vs. Re*Z* dependence shows complexity in the dielectric behavior with interactions between conduction mechanisms at grain boundaries and within grains, with possible additional contributions from other microstructural features that lead to the overlapping of multiple semicircles into a depressed and extended one. The non-ideal behavior of real materials, due to structural heterogeneity, porosity, and a wide distribution of relaxation times caused by the local variation in polarization phenomena at grain boundaries or interfaces, with a power-law response in frequency, cannot be easily described by an ideal capacitor. Instead, a constant phase element (*CPE*) takes into account all of these non-ideal aspects and allows a more adequate description of the material. Thus, the equivalent circuit containing a resistance, *R*_s_, connected in series with two *CPE*//*R* branches intends to describe the dielectric behavior created by individual contributions and interactions between the various processes present in the high-entropy ceramics. More precisely, the first *CPE*_gb_//*R*_gb_ branch is ascribed to the grain boundary capacitance/resistance in the mid-frequency range, and the *CPE*_g_//*R*_g_ branch is ascribed to the geometrical capacitance/resistance of the bulk grains in the high-frequency range. The results are listed in Table 7.

Furthermore, an equivalent capacitance *C* value can be assigned to the *CPE* element in a given frequency range, considering that the impedance of a *CPE* element is given by the equation [90,91]:

(8) $$Z_{CPE}=\frac{1}{Q{\left(j\omega \right)}^{n}}$$

and the impedance expression of a *CPE*//*R* branch in the equivalent circuit is described by the equation:

(9) $$Z=\frac{R}{1+RQ{\left(j\omega \right)}^{n}}$$

in which *Q* and *n* (0 < *n* < 1, *n* = 1 for an ideal capacitor when *Q* = *C*) represent the numerical value of the admittance (1/|*Z*|) at *ω* = 1 rad/s and the exponent of the *CPE*, *j* is the imaginary unit, *ω* is the angular frequency, and *R* is the resistance corresponding to the *CPE* in the *CPE*//R branch. For each *CPE*_gb_ and *CPE*_g_, the corresponding equivalent capacitances *C*_gb_ and *C*_g_ were calculated and are listed in Table 7.

The equivalent circuit model fits well with the experimental data and the Nyquist plots generated by the model are comparable with those obtained experimentally. In the *CPE*//*R* branch associated with grain boundaries, both the resistance *R*_gb_ and *C*_gb_ decrease with increasing <*GS*>. This behavior suggests that interfaces become more conductive (resistance decreases) and that less heterogeneity or defects are present to contribute to interface polarization (equivalent capacitance decreases). As the grain size increases, the charge storage capacity at grain boundaries changes and the grain boundary strength decreases due to improved connectivity between the grains. This also leads to a reduction in the interfacial polarization and in the energy barriers to the movement of charge carriers. The values of the exponent *n*_gb_ determined in the branch related to the boundaries are worth a closer look. *n*_gb_ = 0.68 indicates a significant heterogeneity at a small grain size, which decreases as the grain size increases and the *CPE* behavior becomes closer and closer to that of an ideal capacitor. Conversely, a decrease in resistance, *R*_g_, along with an increase in equivalent capacity, *C*_g_, can be observed in the *CPE*//*R* branch associated with grains. As the <*GS*> increases, the bulk charge storage capacity increases as a consequence of an improvement in the structural ordering, which allows a greater accumulation of charge inside the grains, and thus, an increase in the bulk polarization. The exponent *n*_g_ is close to 1 in ceramics with the lowest grain size and stabilizes to 1 with increasing <*GS*>, indicating structural and chemical uniformity within the grains. *CPE*_g_ acts as a pure capacitance in this case, suggesting an ideal dielectric behavior and the absence of significant heterogeneities or defects, which is reflected in a well-defined dielectric polarization. *R*_s_, *R*_gb_, and *R*_g_ values decrease with increasing grain size (Figure 14c) due to a lower density of grain boundary regions, which favors the migration of charge carriers. This also supports the conductivity data.

To conclude, with increasing <*GS*>, better structural uniformity is achieved, with a significant impact on the electrical properties. The enhanced structural uniformity is reflected in the transition to a more ideal dielectric behavior leading to improved grain boundary conductivity and ideal capacitive response in the bulk.

## 4. Conclusions

In this study, novel high-entropy perovskite (Bi_0.2_K_0.2_Ba_0.2_Ca_0.2_Sr_0.2_)TiO_3_ powders and related ceramics were prepared by a modified Pechini route and subsequent conventional sintering. The detailed characterization showed that the starting powder consists of nano-sized particles with a narrow particle size distribution, an average particle size of 16–18 nm, and a homogeneous distribution of the cation species. The processing of ceramics at different temperatures between 900 °C and 1000 °C leads to obtaining a mixture of cubic and tetragonal polymorphs and grain sizes of 35 to 50 nm. Increasing sintering temperature determines the grain size increase, which favors the tetragonal modification but also affects the densification because of the volatilization processes related to Bi^3+^ and K^+^ species. The measured dielectric properties depict a ferroelectric–relaxor crossover with diffuse low-permittivity maxima dependent on the microstructure, especially on the grain size, which in turn favors the tetragonal modification. The thermally stable dielectric response, with low values of permittivity and dielectric losses over a large temperature range, are critical requirements for microwave devices. Therefore, this work suggests that tailoring the microstructure by using suitable synthesis procedures in high-entropy ceramics strongly influences their functional properties, contributing to an enlargement in the field of their use in electronic applications, especially those involving miniaturization and a high integration degree.

## Figures and Tables

**Table 1:** *A*-site high-entropy perovskite compositions, configurational entropy, average *A*-site ionic radius, *A*-site atomic size disorder factor, and Goldschmidt tolerance factor.

	Composition	Reference	*S_config_*	<*R_A_*>	*δ*(*R_A_*) (%)	*t*
Balanced charge	(Bi_0.2_Li_0.2_Ba_0.2_Sr_0.2_Pb_0.2_)TiO_3_	[44]	1.61R	1.434	21.15	0.956
	(Bi_0.2_Ag_0.2_Ba_0.2_Sr_0.2_Pb_0.2_)TiO_3_	[44]	1.61R	1.512	12.02	0.985
	(Bi_0.2_Na_0.2_Ba_0.2_Sr_0.2_Ca_0.2_)TiO_3_	[44]	1.61R	1.53	9.33	0.991
	(Bi_0.2_Na_0.2_Ba_0.2_Ca_0.2_Sr_0.2_)TiO_3_	[55]	1.61R	1.53	9.33	0.991
	(Bi_0.2_Na_0.2_Sr_0.2_Ba_0.2_Ca_0.2_)TiO_3_	[43]	1.61R	1.53	9.33	0.991
	(Bi_0.2_Na_0.2_Ba_0.2_Sr_0.2_Pb_0.2_)TiO_3_	[44]	1.61R	1.56	9.28	1.002
	(Bi_0.2_K_0.2_Ba_0.2_Ca_0.2_Sr_0.2_)TiO_3_	This work	1.61R	1.58	11.03	1.009
	(Bi_0.2_K_0.2_Ba_0.2_Sr_0.2_Pb_0.2_)TiO_3_	[44]	1.61R	1.61	10.39	1.020
Unbalanced charge	(Ba_0.2_Na_0.2_Mg_0.2_La_0.2_Bi_0.2_)TiO_3_	[34]	1.61R	1.424	16.96	0.953
	(Ba_0.2_Na_0.2_K_0.2_Mg_0.2_Bi_0.2_)TiO_3_	[42]	1.61R	1.446	23.41	0.961
	(Bi_0.2_Na_0.2_K_0.2_Li_0.2_Sr_0.2_Ca_0.2_)TiO_3_	[29]	1.61R	1.452	16.98	0.963
	Na_0.30_K_0.07_Ca_0.27_La_0.18_Ce_0.21_TiO_3_	[56]	1.54R	1.465	15.56	0.968
	(Ba_0.2_Na_0.2_Ca_0.2_Sm_0.2_Bi_0.2_)TiO_3_	[42]	1.61R	1.49	10.13	0.977
	(Bi_0.2_Na_0.2_K_0.2_Ba_0.2_Ca_0.2_)TiO_3_	[57]	1.61R	1.57	11.17	1.006
	(Bi_0.2_Na_0.2_K_0.2_Li_0.2_Ca_0.2_Mg_0.2_)TiO_3_	[29]	1.93R	1.638	25.44	1.030
	(Bi_0.2_Na_0.2_K_0.2_Li_0.2_Sr_0.2_Mg_0.2_)TiO_3_	[29]	1.93R	1.658	25.74	1.037
	(Bi_0.2_Na_0.2_K_0.2_Li_0.2_Ba_0.2_Mg_0.2_)TiO_3_	[29]	1.93R	1.692	26.71	1.050
	(Bi_0.2_Na_0.2_K_0.2_Li_0.2_Ca_0.2_Pb_0.2_)TiO_3_	[29]	1.93R	1.758	23.23	1.074
	(Bi_0.2_Na_0.2_K_0.2_Li_0.2_Sr_0.2_Pb_0.2_)TiO_3_	[29]	1.93R	1.778	23.29	1.081
	(Bi_0.2_Na_0.2_K_0.2_Li_0.2_Ba_0.2_Ca_0.2_)TiO_3_	[29]	1.93R	1.782	23.81	1.082
	(Bi_0.2_Na_0.2_K_0.2_Li_0.2_Ba_0.2_Sr_0.2_)TiO_3_	[29]	1.93R	1.802	23.79	1.089
	(Bi_0.2_Na_0.2_K_0.2_Li_0.2_Ba_0.2_Pb_0.2_)TiO_3_	[29]	1.93R	1.812	23.87	1.093

**Table 2:** Structural features obtained for the (Bi_0.2_K_0.2_Ba_0.2_Sr_0.2_Ca_0.2_)TiO_3_ precursor powders.

	Cubic, Pm-3m (49.4%)	Tetragonal, P4mm (45.7%)	Orthorhombic, Pnma (3.1%)	Bi_2_O_3_ (1.8%)
COD #	96-900-6865	96-151-3253	96-900-2804	96-101-0313
Crystal system	Cubic	Tetragonal	Orthorhombic	Tetragonal
*a* (Å)	3.907 ± 0.002	3.999 ± 0.041	5.378 ± 0.002	-
*b* (Å)	3.907 ± 0.002	3.999 ± 0.041	7.382 ± 0.011	-
*c* (Å)	3.907 ± 0.002	4.037 ± 0.082	5.421 ± 0.002	-
*V* (Å^3^)	59.64	64.58	215.18	-
*R*_exp_	17.17			
*R*_p_	9.21			
*R*_wp_	14.43			
χ^2^	0.71			
Average crystallite size <*D*> (nm)	14.24 ± 9.12	1.93 ± 0.26	25.66 ± 6.23	

**Table 3:** Chemical composition estimated from XRF measurements for the main elements of (Bi_0.2_K_0.2_Ba_0.2_Sr_0.2_Ca_0.2_)TiO_3_ powder calcined at 900 °C.

Element	Wt%	Est. Error
Ti	32.03	0.23
Bi	25.79	0.22
Ba	16.75	0.19
Sr	10.82	0.16
Ca	6.54	0.12
K	5.84	0.12

**Table 4:** Structural and microstructural features obtained for conventionally sintered ceramic materials at temperatures of 900, 950, and 1000 °C for 4 h.

Processing Parameters	900 °C/4 h		950 °C/4 h		1000 °C/4 h	
Crystal system	Tetragonal	Cubic	Tetragonal	Cubic	Tetragonal	Cubic
Phase amount (%)	54.3	45.7	66.6	33.4	73.7	26.3
*a* (Å)	3.819 ± 0.002	3.983 ± 0.001	3.912 ± 0.003	3.980 ± 0.001	3.936 ± 0.007	3.967 ± 0.001
*c* (Å)	3.964 ± 0.005	3.983 ± 0.001	3.938 ± 0.006	3.980 ± 0.001	3.938 ± 0.015	3.967 ± 0.001
*c*/*a*	1.038	1.000	1.006	1.000	1.001	1.000
*V* (Å^3^)	60.87	63.19	60.28	63.020	60.99	62.41
ρ_t_ (g/cm^3^)	5.35		5.37		5.36	
ρ_a_ (g/cm^3^)	4.65		4.41		4.17	
ρ_r_ (%)	86.89		82.09		77.77	
*R*_exp_	6.90		6.94		7.03	
*R*_p_	5.38		5.43		5.28	
*R*_wp_	7.48		7.53		7.01	
χ^2^	1.18		1.18		0.99	
Average crystallite size <*D*> (nm)	5.58 ± 2.23	40.31 ± 14.05	6.38 ± 1.60	28.05 ± 9.57	7.93 ± 3.84	28.53 ± 15.25
Grain size <*GS*> (nm)	35.93 ± 17.59		40.82 ± 10.11		50.53 ± 13.94	

**Table 5:** Experimental and fit parameters for the high-entropy ceramics.

Samples	<*GS*> (nm)	*ε′*_m_	*T*_m_	Δ	*ξ*	*ε′* (RT)	Tan *δ* (RT)
BiKBSCT-900	50.53	41	249	690	1.58	40	0.0074
BiKBSCT-950	40.82	58	266	678	1.53	58	0.0084
BiKBSCT-1000	35.93	157	276	615	1.49	156	0.088

**Table 6:** The exponent values obtained by fitting the frequency dependence of the ac conductivity with Jonscher’s double power law at three different fixed temperatures for the high-entropy ceramics under investigation.

Sample	Measuring Temperature					
	100 K		300 K		500 K	
	*n*	*m*	*n*	*m*	*n*	*m*
BiKBSCT-900	0.97	1.97	0.98	1.97	0.33	2
BiKBSCT-950	0.97	1.96	0.98	1.96	0.32	2
BiKBSCT-1000	0.98	1.96	0.89	1.92	0.3	1.98

**Table 7:** Values of the circuit elements.

Sample	*R*_s_	*Q*_gb_	*n*_gb_	*R*_gb_	*Q*_g_	*n*_g_	*R*_g_	*C*_gb_	*C*_g_
BiKBSCT-900	703	8.18 × 10^−8^	0.68	164,350	1.61 × 10^−11^	0.999	3.16 × 10^10^	9.67 × 10^−9^	1.61 × 10^−11^
BiKBSCT-950	593	3.44 × 10^−8^	0.73	150,600	1.88 × 10^−11^	1	9.64 × 10^9^	4.24 × 10^−9^	1.88 × 10^−11^
BiKBSCT-1000	364	3.0 × 10^−8^	0.74	144,680	2.78 × 10^−11^	1	8.03 × 10^9^	4.07 × 10^−9^	2.78 × 10^−11^

## Data Availability

Data is contained within the article.
